# WEB-based GEne SeT AnaLysis Toolkit (WebGestalt): update 2013

**DOI:** 10.1093/nar/gkt439

**Published:** 2013-05-23

**Authors:** Jing Wang, Dexter Duncan, Zhiao Shi, Bing Zhang

**Affiliations:** ^1^Department of Biomedical Informatics, ^2^Advanced Computing Center for Research and Education and ^3^Department of Cancer Biology, Vanderbilt University, Nashville, TN 37203, USA

## Abstract

Functional enrichment analysis is an essential task for the interpretation of gene lists derived from large-scale genetic, transcriptomic and proteomic studies. WebGestalt (WEB-based GEne SeT AnaLysis Toolkit) has become one of the popular software tools in this field since its publication in 2005. For the last 7 years, WebGestalt data holdings have grown substantially to satisfy the requirements of users from different research areas. The current version of WebGestalt supports 8 organisms and 201 gene identifiers from various databases and different technology platforms, making it directly available to the fast growing omics community. Meanwhile, by integrating functional categories derived from centrally and publicly curated databases as well as computational analyses, WebGestalt has significantly increased the coverage of functional categories in various biological contexts including Gene Ontology, pathway, network module, gene–phenotype association, gene–disease association, gene–drug association and chromosomal location, leading to a total of 78 612 functional categories. Finally, new interactive features, such as pathway map, hierarchical network visualization and phenotype ontology visualization have been added to WebGestalt to help users better understand the enrichment results. WebGestalt can be freely accessed through http://www.webgestalt.org or http://bioinfo.vanderbilt.edu/webgestalt/.

## INTRODUCTION

High-throughput genomic, transcriptomic and proteomics technologies have transformed biological research by enabling comprehensive investigations of biological systems. Analysis of the resulted genome-scale data typically yields lists of interesting genes or proteins. How to translate the identified gene sets into a better understanding of the underlying biological themes has become a fundamental need in biological research. In response to this critical need, in the 2005 NAR (Nucleic Acids Research) Web Server Issue, we presented WebGestalt (WEB-based GEne SeT AnaLysis Toolkit) (1), one of the first software applications that integrate functional enrichment analysis and information visualization for the management, information retrieval, organization, visualization and statistical analysis of large sets of genes. Ever since its publication, the tool has been widely used in large-scale genetic, transcriptomic and proteomic studies, with more than 400 citations reported by Google Scholar as of the time of writing.

**Figure 1.**
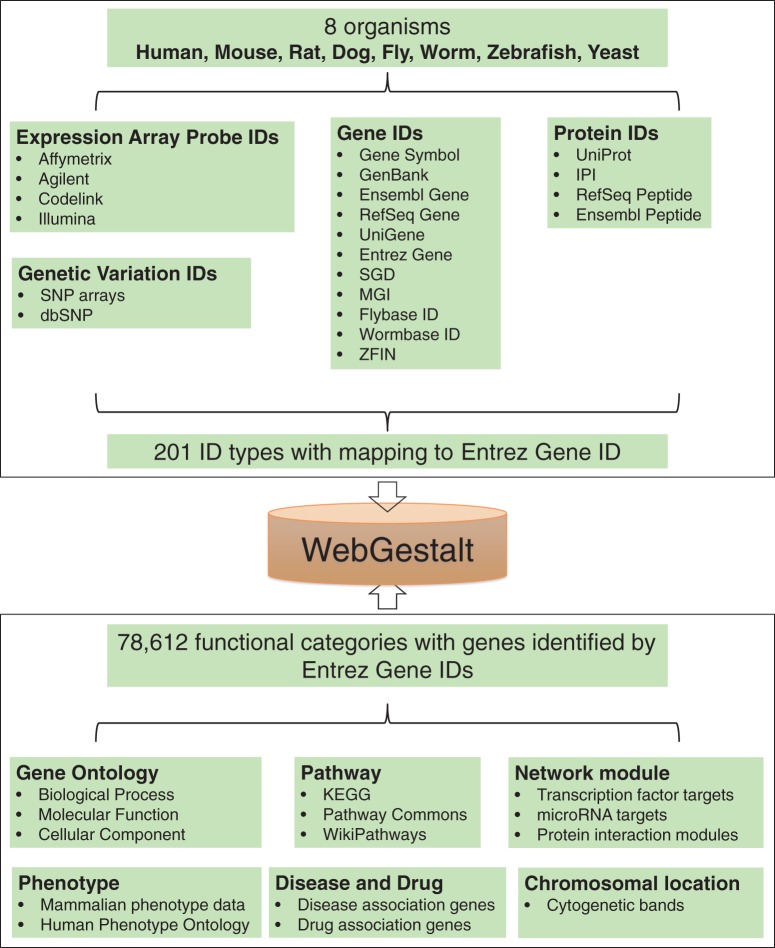
Summary of organisms, gene identifiers and functional categories supported by WebGestalt.

During the last 7 years, with the rapid development of high-throughput technologies, omics data from diverse experimental platforms for human and other model organisms are accumulating exponentially. To satisfy the needs of biologists from different research areas, enrichment analysis tools should be directly applicable to data generated from different platforms and organisms. Thus, WebGestalt and some related tools (e.g. DAVID (2), FatiGO (3) and g:Profiler (4)) are constantly updated to support more organisms and platforms. The current version of WebGestalt supports 8 organisms and 201 gene identifiers from various databases and different experimental platforms (Figure 1).

Another important consideration for enrichment analysis tools is to update and expand the collection of functional categories. Most of these tools integrate multiple centrally curated functional databases, such as Gene Ontology (GO) (5), KEGG (6), Pathway Commons (7) and MSigDB (8). In contrast to centrally curated databases, open, collaborative platforms that allow for broader participation by the entire biology community, such as WikiPathways (9), have become a new model for community-based curation of biological data. This new model enhances and complements ongoing effort of centrally curated databases and holds great potential to significantly enhance our knowledge on functional categories. In addition to manual curation, computational analysis represents an alternative approach for building functional categories. For example, motif gene sets derived from comparative genomic analysis of conserved *cis*-regulatory motifs have been included in MSigDB. Recently, it has been shown that large gene and protein interaction networks can be used to infer ontologies whose coverage and power are comparable to those manually curated by the GO consortium (10). We have also built GLAD4U (11), a tool that derives and prioritizes gene lists from PubMed literature for a user provided search term, which opens the possibility to create gene sets related to concepts such as diseases and drugs. Despite their obvious importance, disease and drug-related gene sets are scarce in the curated databases. For example, the average number of genes for each disease in the OMIM (Online Mendelian Inheritance in Man) database (12) is 2.7, making it unsuitable for enrichment analysis. Thus, computationally derived gene sets may serve as an important data source to enhance functional enrichment analysis. The new version of WebGestalt integrates data from centrally and publicly curated databases as well as computational analyses, leading to a total of 78 612 functional categories (Figure 1).

In addition to significant data expansion, WebGestalt has also improved user friendliness and added new visualization features that help users better understand the enrichment results. WebGestalt can be freely accessed through http://www.webgestalt.org or http://bioinfo.vanderbilt.edu/webgestalt/.

## NEW DATA

### Gene identifier

As shown in Table 1, the old version of WebGestalt only supported seven gene identifiers from a few public databases for human and mouse, including Entrez Gene ID, Gene Symbol, RefSeq for DNA, RefSeq for protein, Unigene, Ensemble ID and Uniprot ID. In contrast, the new version of WebGestalt not only supports identifiers from more public databases but also recognizes identifiers from major high-throughput platforms for human and seven important model organisms including mouse, rat, worm, fly, yeast, dog and zebrafish. Using human as an example, WebGestalt has increased the number of identifiers from public databases to 13. Meanwhile, WebGestalt has added 35, 4, 5 and 1 identifiers from Affymetrix, Agilent, Illumina and Codelink microarrays, respectively. Although identifiers from gene expression arrays are typically supported by enrichment analysis tools, those from SNP arrays are less well supported. It is increasingly recognized that pathway-based enrichment analysis can greatly complement the single-SNP-based analysis in understanding genetic determinants of common diseases and providing insights into the pathway dysregulation of complex diseases (13,14). WebGestalt has added 21 identifiers from commonly used human SNP array platforms, allowing easy translating of SNP data into biological insights. All 201 supported identifiers for the 8 organisms can be found in Supplementary Table S1. These identifiers will be mapped to Entrez Gene IDs for enrichment analysis in WebGestalt (Figure 1).

**Table 1.** Significantly increased data coverage in WebGestalt

	Organism	Gene identifier	Functional category
Old version	*Homo sapiens*	7	Gene Ontology
	*Mus musculus*	7	Pathway
New version	*Homo sapiens*	61	Gene Ontology Pathway Network Phenotype Disease and Drug Chromosomal location
	*Mus musculus*	43	
	*Rattus norvegicus*	27	
	*Canis familiaris*	12	
	*Drosophila melanogaster*	13	
	*Danio rerio*	18	
	*Caenorhabditis elegans*	13	
	*Saccharomyces cerevisiae*	14	

### Functional category

The old version of WebGestalt only included two types of functional categories (Table 1), whereas the new version covers six types (GO, Pathway, Network, Phenotype, Disease and Drug, and Chromosomal location) (Table 2). For the pathway collection, in addition to the KEGG database, WebGestalt has added data from Pathway Commons and WikiPathways. Because Pathway Commons collects and integrates nine centrally curated biological pathway databases and WikiPathways is a primary source for open community-based curation, adding these two resources has significantly increased the coverage of pathways in WebGestalt. Gene–phenotype association is one of the most important aspects biology. Therefore, we have added phenotype-associated gene sets defined by Mammalian Phenotype Ontology (15) and Human Phenotype Ontology (16) to WebGestalt. WebGestalt has also included gene sets defined by cytogenetic bands, which can help identify alterations related to chromosomal deletions or amplifications, dosage compensation and other regional effects.

**Table 2.** Detailed information on the functional categories supported by WebGestalt

Type	Database	Method	No. of categories^a^	No. of organisms^b^	Data source
Gene Ontology	Biological process	CC	24 278	8	Gene Ontology (http://www.geneontology.org/)
	Cellular component	CC	3079	8	
	Molecular function	CC	9508	8	
Pathway	KEGG	CC	390	8	R package KEGG.db
	Pathway Commons	CC	1651	1	http://www.pathwaycommons.org/pc/
	WikiPathways	PC	1018	8	http://www.wikipathways.org/index.php/WikiPathways
Network	Hierarchical protein interaction network module	CA	1993	2	HPRD (http://www.hprd.org/)
					BioGrid (http://thebiogrid.org/)
					BOND (http://bond.unleashedinformatics.com/)
					DIP (http://dip.doe-mbi.ucla.edu/dip/)
					IntAct (http://www.ebi.ac.uk/intact/)
					MINT (http://mint.bio.uniroma2.it/mint/)
					Reactome (http://www.reactome.org/)
	MicroRNA target	CA	884	4	MSigDB (http://www.broadinstitute.org/gsea/msigdb)
	Transcription factor target	CA	2460	4	MSigDB (http://www.broadinstitute.org/gsea/msigdb)
Phenotype	Phenotype	CC	19 023	2	Mammalian Phenotype Ontology (http://www.informatics.jax.org)
					Human Phenotype Ontology (http:www.human-phenotype-ontology.org)
Disease and Drug	Disease	CA	2286	1	GLAD4U (http://bioinfo.vanderbilt.edu/glad4u)
	Drug	CA	758	1	GLAD4U (http://bioinfo.vanderbilt.edu/glad4u)
Chromosomal location	Cytogenetic band	CC	11 284	4	Entrez Gene (http://www.ncbi.nlm.nih.gov/gene)

^a^The number of categories in each database.

^b^The number of organisms supported by the database.

CC, centrally curated; PC, publicly curated; CA, computational analysis.

One important addition to the new version of WebGestalt includes functional categories defined by network modules, comprising protein–protein interaction network modules and regulatory modules. The former is a recent and unique addition to WebGestalt through computational network analysis. Although a few enrichment analysis tools support protein–protein interaction network-based enrichment analysis, they typically rely on gene sets derived from network decomposition at a single level without considering the hierarchical structure of the network. However, it is well-known that hierarchical organization is a critical intrinsic property of complex systems including biological networks (17). Recent works from the Ideker group (10) suggest that gene networks embed hierarchical structure that is consistent with GO and can extend beyond GO. Along the same line, WebGestalt has added functional categories defined by hierarchical protein interaction network modules. Specifically, for the human network, we combined from seven curated databases (Table 2) all protein–protein interaction data with at least one publication support to build an integrated protein–protein interaction network. For the mouse network, because the seven databases only contained a limited number of interactions, we first used an ortholog-based method (18) to infer mouse interactions from curated human interactions and then combined them with curated mouse interactions. We implemented—with some modifications—the method previously published by Sales-Pardo *et al.* (19) to identify hierarchical modules from the integrated networks. Although a standard hierarchical clustering is able to reveal hierarchical structure of a network, it does not specify relevant hierarchical levels and modules at different scales. Moreover, it does not assess the statistical significance of the modular organization of a network. Our implementation directly addresses these limitations. Here we briefly describe our network clustering method. A detailed description of our implementation will be included in a separate manuscript. First, using the random walk-based walktrap algorithm (20), we identify the best partition of the network by maximizing the modularity score (21); Secondly, we use the edge switching algorithm (22) to generate 1000 random networks with the same attributes as the protein–protein interaction network and then identify the best partition and corresponding modularity score for each random network; Thirdly, if the modularity score for the interaction network is significantly higher than those for the 1000 random networks (*P* < 0.05), the interaction network is considered to have a modular organization and is divided into sub-networks (modules) according to the identified best partition. To reveal network modules at different hierarchical levels, we repeat the above three steps iteratively for each sub-network until none of them show a modular organization. Using this method, we identified 987 and 1006 hierarchical modules for the human and mouse protein–protein interaction networks, respectively. Eighty percentage of the modules were enriched for at least one GO term (Fisher’s exact test, FDR < 0.05), suggesting high functional relevance of the network-derived modules. More importantly, network modules without GO enrichment may represent functional categories that are not well captured by existing knowledge. Gene sets defined by these protein interaction network modules have been added to WebGestalt. WebGestalt has also added regulatory modules defined as sets of genes sharing common transcription factor or microRNA binding sites, which were inferred from comparative genomic analysis and made available through MSigDB (8).

Another unique addition to the new version of WebGestalt includes functional categories defined by gene sets associated with diseases or drugs, inferred using our recently published software GLAD4U (11). We first downloaded the disease and drug terms from PharmGKB (23) and then used GLAD4U to derive a gene list for each term. Specifically, for a disease or drug term, GLAD4U queries the term in the MEDLINE database using the eSearch application programming interface (API) developed by the National Center for Biotechnology Information (NCBI), retrieves the corresponding publications, identifies genes associated with the publications based on the gene-to-publication link table provided by Entrez Gene, and returns genes that are significantly associated with the query term. Among all the terms downloaded from PharmGKB, GLAD4U returned at least 5 genes for 2286 disease terms and 758 drug terms. These disease or drug-associated gene sets have been added to WebGestalt. To demonstrate the usefulness of these new gene sets, we uploaded a recently published gene expression signature for colorectal cancer prognosis (24), which includes 487 genes inferred by prioritizing candidate genes identified from genomic and transcriptomic studies on a protein–protein interaction network. The list of all 11 521 genes in the network was used a reference set for the enrichment analysis. As expected, the disease association analysis in WebGestalt correctly identified ‘Colorectal Neoplasms’ as the most enriched disease term (FDR = 7.77e-9). Interestingly, the drug association analysis also correctly identified ‘Fluorouracil’ (FDR = 0.043), a primary drug used in the treatment of colorectal cancer.

## NEW FEATURES

**Figure 2.**
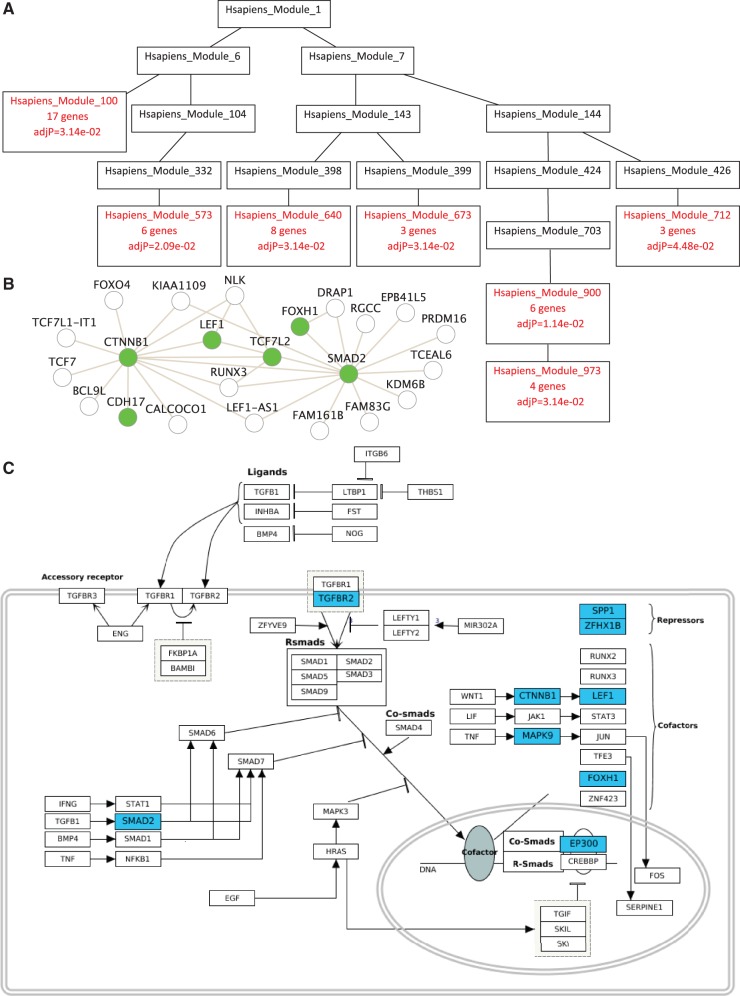
New visualization features in WebGestalt. (**A**) Visualization of the enriched protein interaction network modules in a DAG. (**B**) Visualization of input genes and their direct neighbors in an enriched module using a node-link diagram. (**C**) Visualization of input genes in an enriched pathway from the WikiPathways database.

Visualization features, such as the enriched DAG (directly acyclic graph) for revealing the hierarchical relationship of enriched GO terms, have been the most appreciated features in WebGestalt according to feedback from our users. In the updated version, enriched DAG visualization has been applied to the phenotype enrichment results for visualizing the hierarchical relationship of enriched phenotype terms. Because our newly defined protein interaction network modules also have a hierarchical organization, we have extended the enriched DAG visualization to present results from network module enrichment analysis (Figure 2A). The enriched DAG shows enriched network modules in red and their non-enriched parents in black. As shown in Figure 2A, enriched modules could be found at different hierarchical levels. Therefore, using only gene sets derived from a single-level decomposition of a network may miss important functional information encoded in the network. Unlike GO categories in which relationship among genes is not defined, relationship among genes in a network module is well-defined by the network graph. To reveal this additional level of information, WebGestalt uses the Cytoscape Web (25) plug in to visualize in a network graph the input genes (in green) and their direct neighbors (in white) in the enriched modules (Figure 2B). For enriched KEGG pathways and WikiPathways, WebGestalt has implemented methods to call APIs provided by corresponding resources to view the pathway maps and highlight input genes in the maps (Figure 2C).

In addition to new visualization features, WebGestalt has added five methods for the multiple-test correction of enrichment *P-*values. Because the number of enriched categories is difficult to predict, WebGestalt has also added a ‘Top10’ feature to provide users a quick overview of the 10 most enriched categories and their enrichment scores, which can help users make decision on an appropriate significance level cutoff for more detailed investigation of the data. Finally, WebGestalt has removed the login requirement and has provided options for downloading the analysis results for archiving and sharing purposes.

## CONCLUSION

The new version of WebGestalt has significantly increased the coverage of organisms, gene identifiers and functional categories, has enabled enrichment analysis against some unique data sources including the publicly curated WikiPathways database, hierarchical network modules, phenotype ontology, and disease and drug-associated gene sets, and has implemented new visualization features for presenting pathway, phenotype and network enrichment analysis results. As shown in Table 3, many unique features allow WebGestalt to maintain strong competitiveness among a large number of software applications related to functional enrichment analysis (26). However, WebGesalt is still far from perfect. For example, DAVID contains information about protein domains, Swiss-Prot keywords and Panther pathways, which is not included in WebGestalt. Thus, in the future, we will continuously update WebGestalt to support more gene identifiers and curated functional categories, develop and adopt novel computational methods to define new functional categories, and improve the intuitiveness and friendliness of the user interface.

**Table 3.** Features unique to WebGestalt or shared with other tools^a^

	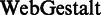	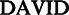	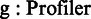	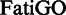
Multiple-organism support	✓	✓	✓	✓
Gene identifier				
Public database	✓	✓	✓	✓
Affymetrix	✓	✓	✓	✓
Agilent	✓	✓	✓	✓
Codelink	✓		✓	✓
SNP	✓	✓		✓
Functional category				
GO	✓	✓	✓	✓
KEGG	✓	✓	✓	✓
Pathway Commons	✓	L^b^	L	L
WikiPathways	✓			
Hierarchical protein interaction network module	✓			
MicroRNA target	✓			✓
Transcription factor target	✓		✓	✓
Phenotype	✓		L	
Disease	✓	✓		
Drug	✓			
Cytogenetic band	✓	✓		
Visualization				
GO DAG	✓	✓	✓	✓
KEGG visualization	✓	✓	✓	✓
WikiPathways visualization	✓			
Network DAG and node-link diagram	✓			
Phenotype DAG	✓			

^a^The purpose of this table is to distinguish which WebGestalt features are unique to WebGestalt and which of them are available in other tools. Thus, the table only lists features available in WebGestalt. This is not meant to be a comprehensive comparison of enrichment analysis tools, and only three other representative tools are included.

^b^DAVID and FatiGO contain Reactome and Biocarta databases whereas g:Profiler contains Reactome and BIOGRID databases. These databases are a subset of the Pathway Commons database; g:Profiler only contains phenotypes from Human Phenotype Ontology.

## SUPPLEMENTARY DATA

Supplementary Data are available at NAR Online: Supplementary Table 1.

## FUNDING

National Institutes of Health (NIH) [GM088822 to B.Z., MH096972, CA159988]. Funding for open access charge: NIH [R01 GM088822 and P50 MH096972].

*Conflict of interest statement*. None declared.
